# Establishment and validation of nomogram for predicting immuno checkpoint inhibitor related pneumonia

**DOI:** 10.1186/s12890-022-02127-3

**Published:** 2022-09-01

**Authors:** Xiaoqi Li, Fei Lv, Ying Wang, Zhenguang Du

**Affiliations:** 1 Department of Oncology III, Liaoning People’s Hospital, 33 Wenyi Road, Shenhe District, Shenyang, 110022 Liaoning Province China; 2 Department of Oncology, Shengjing Hospital of China Medical University, Shenyang, Liaoning China

**Keywords:** Immune checkpoint inhibitors, Krebs von den Lungen-6 protein, Nomograms, Pneumonia, Pulmonary surfactant-associated protein A

## Abstract

**Objective:**

Cancer is one of the main causes of death worldwide. Although immunotherapy brings hope for cancer treatment, it is also accompanied by immune checkpoint inhibitor-related adverse events (irAEs). Immune checkpoint inhibitor pneumonia (CIP) is a potentially fatal adverse event, but there is still a lack of effective markers and prediction models to identify patients at increased risk of CIP.

**Methods:**

A total of 369 cancer patients treated between 2017 and 2022 with immune checkpoint inhibitors at Shengjing Hospital of China Medical University and Liaoning People's Hospital were recruited for this study. Independent variables were selected by differences and binary logistic regression analysis, and a risk assessment nomogram was constructed for CIP risk. The accuracy and discriminative abilities of the nomogram were evaluated by calibration plots, receiver operating characteristic curves (ROCs) and decision curve analyses (DCAs).

**Results:**

Binary logistic regression analysis showed that smoking history, acute phase proteins [interleukin (IL-6) and C-reactive protein (CRP)], CD8 + T lymphocyte count and serum alveolar protein [surface protein-A (SP-A) and Krebs Von den Lungen-6 (KL-6)] were significantly associated with CIP risk. A nomogram consisting of these variables was established and validated by different analyses.

**Conclusions:**

We developed an effective risk nomogram for CIP prediction in immune-checkpoint inhibitor administrated cancer patients, which will further assist early detection of immunotherapy-related adverse events.

## Background

Cancer is a serious threat to human health and life and is the second most important cause of death after heart disease. In 2022, nearly five million new cancer cases and three million cancer deaths are projected to occur in China [1]. Immune checkpoint inhibitors (ICIs) have changed the pattern of tumour treatment; however, they are accompanied by multisystem ICI-related adverse events (irAEs). irAEs occur in up to 70% of patients administered Programmed Death-(Ligand) 1 inhibitors [2]. Most irAEs, including cutaneous, liver, gastrointestinal, and endocrine adverse events, are controllable. However, some irAEs occurring in the heart, lungs, liver, and nervous system are potentially fatal and life-threatening.

Immune checkpoint inhibitor pneumonia (CIP) is a kind of irAE that occurs in the lungs, and the main lesion is interstitial pneumonia originating in the lower lobes of both lungs. CIP has been reported to have a morbidity rate of 3–5% and a mortality rate of 10–17% and it may be higher in patients with lung cancers [3]. Its main symptoms are often nonspecific, including dyspnoea, cough, fever and chest pain. CIP occurs mainly in the first 6 months after starting treatment [4]. Glucocorticoids can be used for CIP treatment, and are usually effective, but approximately 1/4 of patients eventually relapse [5]. As a serious irAE, CIP has a hidden onset. It is PD-1/PD-L1 dose-independent and lacks effective early predictive and models. After therapy, about one-fourth of CIP patients will develop recurrence [6].

Pulmonary surfactant is an important lipoprotein complex that is mainly produced by alveolar epithelial cells (AECs). Pulmonary surfactant plays an important role in alveolar air exchange and participates in pulmonary immune regulation and antiviral infection. Surfactant protein A (SP-A) and surfactant protein D (SP-D) are large, soluble, hydrophilic proteins that are important components of the pulmonary surfactant. The production of pulmonary surfactant may increase after alveolar epithelial cell injury. However, the markers of alveolar epithelial cell lesions lack specificity and are changed in many diseases, such as systemic sclerosis-associated interstitial lung disease [7] and idiopathic pulmonary interstitial fibrosis [8]. For cancer, *Hasegawa* et al. reported that SP-D inhibited the proliferation and motility of NSCLC by binding to epidermal growth factor receptor (EGFR) directly [9], so serum SP-D levels may predict the response to EGFR-tyrosine kinase inhibitors (EGFR-TKIs) [10].

Krebs von den Lungen-6 (KL-6), which is classified as one of the human Mucin 1 antigens, is a high molecular weight glycoprotein expressed predominantly by AECs, bronchial glandular epithelial cells and bronchial gland cells [11]. KL-6 was initially studied as a cancer biomarker. When the expression of KL-6 in the serum and tumour tissue of patients with non-small cell lung cancer is increased, they have a poor prognosis [12, 13] and worse curative effect of EGFR-TKIs [14]. KL-6 is only expressed at low levels in normal lung tissue and terminal bronchiolar epithelial cells, and the expression of KL-6 is significantly increased in compensatory hyperplasia of AECs after injuries, which could be caused by connective tissue disease (CTD) [15], viral infections such as COVID-19 [16] and chest radiotherapy[17]. KL-6 can be released from regenerated type II AECs and secreted into the bloodstream through the damaged alveolar basement membrane [15].

As an irAE with high mortality, CIP still lacks effective markers and prediction models. In this study, we aimed to identify risk factors and construct nomograms for evaluating the individual risk of CIP. We enrolled a total of 369 PD-1/PD-L1 monoclonal antibody-treated cancer patients as the training cohort and validation cohort. In the training cohort, variables associated with CIP risk in the differential analysis were included in the binary logistic regression analysis to identify independent risk factors, and a nomogram was established based on the variables. Then, we used a calibration curve [18], receiver operating characteristic (ROC) curve analysis and decision curve analysis (DCA) [19] to assess the goodness of fit, accuracy and applicability of the predictive nomogram in the training and validation cohorts.

## Methods

### Patients

A total of 245 immunotherapy-treated cancer patients from Shengjing Hospital (as a training cohort) and 124 patients from Liaoning People's Hospital (as a validation cohort) were enrolled in this study between January 1, 2017, and December 31, 2021, and were followed up until February 28, 2022. All of these patients had a definite pathological diagnosis of cancer and were treated with PD1/PD-L1 monoclonal antibodies. The patients with rheumatologic disease, radiation pneumonitis, drug-induced pneumonia and other pre-existing lung abnormalities were excluded from the study. At the same time, other information from these patients, including demographic characteristics (including age, sex, body mass index (BMI), smoking history), the primary and secondary sites of the tumour and prior oncologic therapy, were also collected.

### Imaging examination

**Fig. 1:**
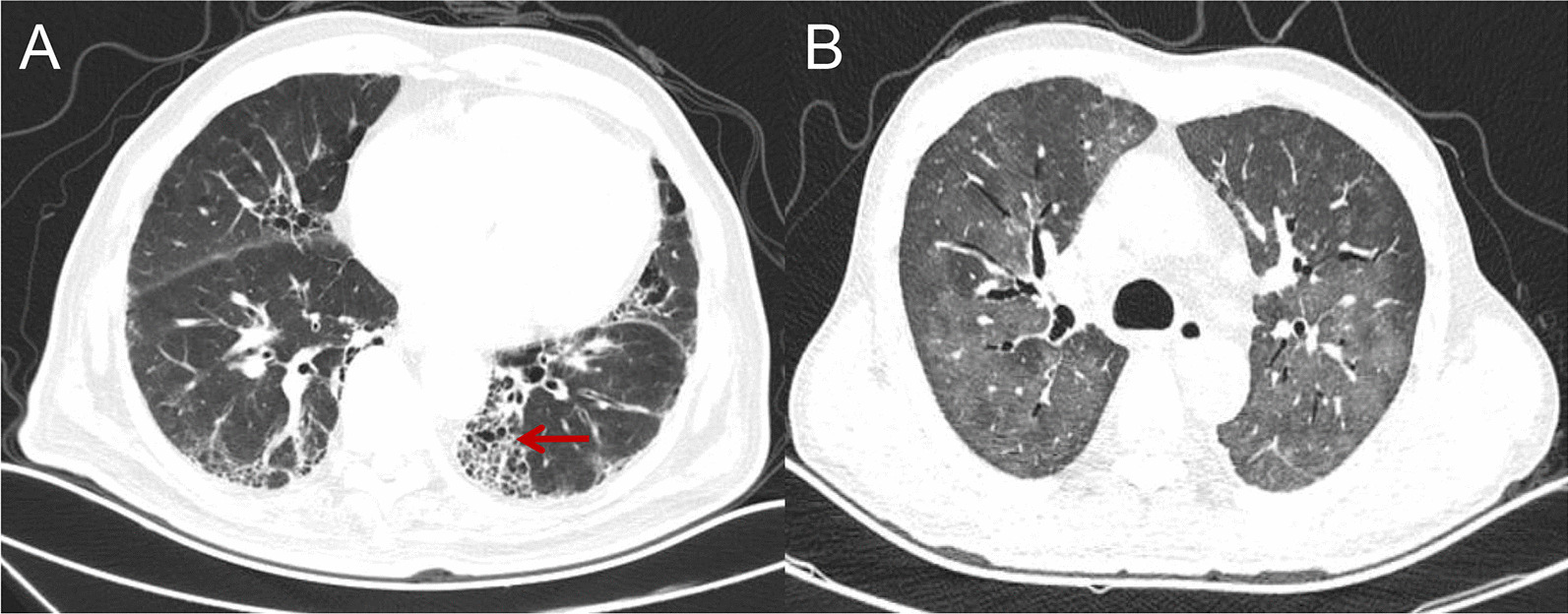
**A** A 59-year-old male patient with colon cancer and lung metastasis was treated with nivolumab combined with CAPEOX. One month after starting treatment, he developed an irritating dry cough and back pain. HRCT showed thickening of the subpleural lobular septum and multiple reticular changes, which were cured after glucocorticoid administration. **B** A 66-year-old male patient with liver cancer with intrahepatic metastasis was treated with toripalimab combined with bevacizumab. Dyspnoea occurred after 6 months of treatment. HRCT showed scattered multiple fuzzy patches in both lungs. The application of glucocorticoid and anti-infection treatment did not work, and the patient ultimately died of respiratory failure

High Resolution Computed Tomography (HRCT), which was obtained with 1.0–1.5 mm at 10-mm intervals at end inspiration from the lung apex to the base, was performed before and every three months during the PD-1/PD-L1 monoclonal antibody administration and reviewed by two independent radiologists (Liu Yang and Li Hongyi) blinded to the diagnosis and clinical course of the patients. Each radiologist described the phenotypic appearance and severity of CIP according to the CT findings: ground-glass attenuation (GGA), consolidation, traction bronchiectasis or honeycombing [20, 21]. At the same time, the radiologists and clinicans distinguished CIP from diseases such as cancerous lymphangitis, pulmonary infection, and alveolar haemorrhage by clinical, labratory and radiology examination (Fig. 1 shows images from 2 typical CIP patients).

### Laboratory examination

Before administration of treatment, blood samples were collected and immediately frozen at − 80 °C until used to obtain a series of laboratory values, including acute phase proteins (IL-6 and CRP), T lymphocyte count and serum alveolar epithelial proteins (including SP-A, SP-D and KL-6).

Taking KL-6 as an example, the serum was centrifuged to obtain the supernatant and diluted at 1: 100 in bovine serum albumin (BSA) to prepare the testing sample. A commercial enzyme-linked immunosorbent assay (ELISA) kit (Abebio, AE36646HU) was used, and the detailed protocol was as described previously [22, 23]. Briefly, the test and standard samples were added to antibody-coated 96-well plates and incubated at 37 °C for 2 h. After washing with PBST, the secondary antibody was added, and then the detection solution was added. Finally, the optical density (OD) values were measured with a multimode plate reader, and the concentration of KL-6 was calculated by a standard curve.

### Statistical analysis

Continuous variables are expressed as the mean ± SD and were compared by Student's t-test, while categorical variables are presented as counts and percentages and were compared using the chi-square test or Fisher’s exact test. Cut-off values of the continuous variables were determined via ROC analysis using MedCalc software. Difference analysis and binary logistic regression analyses were used to analyse the risk factors, and the risk factors with *P* < 0.1 in the difference analysis were included in the binary logistic regression analysis. In addition, variance inflation factor (VIF) values were calculated to measure the degree of multicollinearity among the variables; that is, a VIF of > 5 indicates a high correlation of the variables [24], and at the same time, Kolmogorov–Smirnova and Box–Tidwell transformation were employed for normality and linear checks, respectively.

All statistically significant risk factors in the multivariate analysis were used to construct the nomogram using R statistical software. We used calibration curves to measure the agreement between the predicted and actual outcomes. The predictive ability of the nomogram was assessed according to the area under the curve (AUC) of the ROC curve with an AUC closer to 1.0 indicating better results [25]. The overall area under each probable risk threshold was calculated using DCA. In all analyses, *P* < 0.05 was considered to indicate statistical significance.

## Results

### Clinicopathological characteristics

During the study period, 245 patients from Shengjing Hospital and 124 patients from Liaoning People's Hospital were recruited for this study. The follow-up time in both groups was less than 2 years, which was slightly longer in the training cohort, and 8.40% of all patients suffered from CIP, most of whom were grades 2–3 and received glucocorticoid therapy. One patient died of interstitial pneumonia complicated by severe pulmonary fungal infection, while the other patients were cured. The basic information of the recruited patients is shown in Table 1. EGFR-TKI therapy in the validation cohort was significantly less common than that in the training cohort, while the other variables were not significantly different between the two cohorts, so we excluded this variable from the following analysis.

**Table 1:** Clinical characters of training and validation cohort

	Training cohort	Validation cohort	*P* value
Following time	15	15.07 (11.45–16.32)	0.05
ICI-P	20/245 (8.16%)	11/124 (8.87%)	0.82
Age	66	66.38 (65.01–67.75)	0.78
Gender	110/245 (44.9%)	56/124 (45.16%)	0.96
BMI	21.21 (20.74–21.68)	20.45 (19.85–21.05)	0.85
Smoking history	46/245 (18.78%)	30/124 (24.19%)	0.22
Lung cancer	66/245 (26.94%)	24/124 (19.35%)	0.11
Lung metastasis	71/245 (28.98%)	31/124 (25%)	0.42
Pleural effusion	44/245 (17.96%)	19/124 (15.32%)	0.52
PD-1/PD-L1 Antibody	109/245 (44.49%)	58/124 (46.77%)	0.68
EGFR-TKI	41/245 (16.73%)	6/124 (4.84%)	**< 0.01**
EGFR antibody	32/245 (13.06%)	13/124 (10.48%)	0.47
Gemcitabine	26/245 (10.61%)	12/124 (9.68%)	0.78
Lung surgery	32/245 (13.06%)	11/124 (8.87%)	0.24
Chest radiotherapy	21/245 (8.57%)	16/124 (12.9%)	0.19
Non-1st line therapy	51/245 (20.82%)	24/124 (19.35%)	0.74
Squamous cancer	102/245 (41.63%)	48/124 (38.71%)	0.59
COPD	31/245 (12.65%)	20/124 (16.13%)	0.36
Asthma	23/245 (9.39%)	9/124 (7.26%)	0.49
IL-6 (pg/ml)	39.32 (36.28–42.36)	40.34 (35.76–44.91)	0.71
CRP (mg/L)	81.81 (76.72–86.89)	82.75 (75.17–90.33)	0.84
CD3 + T (/ul)	1924.29 (1872.2–1976.38)	1911.72 (1839.23–1984.21)	0.78
CD4 + T (/ul)	879.9 (826.71–933.09)	878.63 (803.66–953.6)	0.98
CD8 + T (/ul)	651.44 (599.12–703.75)	650.07 (576.59–723.55)	0.98
SP-A (ng/ml)	61.44 (60.04–62.85)	62.48 (60.32–64.63)	0.41
SP-D (ng/ml)	259.64(255.46–263.82)	262.32 (256.67–267.97)	0.46
KL-6 U/ml)	398.16 (381.57–414.75)	412.6 (389.79–435.42)	0.31
FVC (%)	68.56 (67.65–69.47)	69.48 (68.13–70.83)	0.25
Total	245	124	

Number in bold represents *P* < 0.05

*ICI-P* immune checkpoint inhibitor related pneumonia, *BMI* body mass index, *PD-(L)1* programmed death (ligand) 1, *EGFR-TKI* epidermal growth factor receptor-tyrosine kinase inhibitor, *COPD* chronic obstructive pulmoriary disease, *IL-6* interleukin-6, *CRP* C-reaction protein, *SP-A* sufactant protein A, *KL-6* Krebs Von den Lungen-6, *FVC* forced vital capacity

### Difference analysis

As shown in Table 2, we found that smoking history, pleural effusion, number of treatment lines, acute phase proteins (IL-6 and CRP), CD8 + T lymphocyte count and serum alveolar epithelial proteins (including SP-A, SP-D and KL-6) were different between the CIP and non-CIP groups in the training cohort (*P* < 0.1). Therefore, we included these variables in the logistic regression analyses.

**Table 2:** Difference analysis of variables in training cohort

	ICI-P	Non ICI-P	*P* value
Age			
< 60	15/20 (75.00%)	162/225 (72.00%)	0.77
≥ 60	5/20 (25.00%)	63/225 (28.00%)	
Gender			
Male	9/20 (45.00%)	102/225 (45.33%)	0.98
Female	11/20 (55.00%)	123/225 (54.67%)	
BMI			
< 25	1/20 (5.00%)	26/225 (11.56%)	0.37
≥ 25	19/20 (95.00%)	199/225 (88.44%)	
Smoking history			
Yes	13/20 (65.00%)	33/225 (14.67%)	**< 0.01**
No	7/20 (35.00%)	192/225(85.33%)	
Lung cancer			
Yes	4/20 (20.00%)	62/225 (27.56%)	0.47
No	16/20 (80.00%)	163/225 (72.44%)	
Lung metastasis			
Yes	7/20 (35.00%)	64/225 (28.44%)	0.54
No	13/20 (65.00%)	161/225 (71.56%)	
Pleural effusion			
Yes	8/20 (40.00%)	36/225 (16%)	**< 0.01**
No	12/20 (60.00%)	189/225 (84%)	
PD-1/PD-L1			
PD-1	11/20 (55.00%)	98/225 (43.56%)	0.32
Antibody			
PD-L1	9/20 (45.00%)	127/225 (56.44%)	
EGFR antibody			
Yes	1/20 (5.00%)	31/225 (13.78%)	0.26
No	19/20 (95.00%)	194/225 (86.22%)	
Gemcitabine			
Yes	2/20 (10.00%)	24/225 (10.67%)	0.77
No	18/20 (90.00%)	201/225 (89.33%)	
Lung surgery			
Yes	1/20 (5.00%)	31/225 (13.78%)	0.26
No	19/20 (95.00%)	194/225 (86.22%)	
Chest radiotherapy			
Yes	0/20 (0.00%)	21/225 (9.33%)	0.31
No	20/20 (100.00%)	204/225 (90.67%)	
Therapy line			
Non 1st	12/20 (60.00%)	39/225 (17.33%)	**< 0.01**
1st	8/20 (40.00%)	186/225 (82.67%)	
Squamous cancer			
Yes	11/20 (55.00%)	91/225 (40.44%)	0.27
No	9/20 (45.00%)	124/225 (59.56%)	
COPD			
Yes	4/20 (20.00%)	27/225 (12%)	0.51
No	16/20 (80.00%)	198/225 (88%)	
Asthma			
Yes	2/20 (10.00%)	21/225 (9.33%)	0.76
No	18/20 (90.00%)	204/225 (90.67%)	
IL-6 (pg/ml)	55.12 (45.13–65.11)	39.32 (36.27–42.37)	**< 0.01**
CRP (mg/L)	101.07 (77.36–124.79)	80.1 (74.94–85.25)	**0.03**
CD3 + T (/ul)	1863.65 (1676.71–2050.59)	1924.29 (1871.97–1976.62)	0.51
CD4 + T (/ul)	884.45 (718.67–1050.23)	879.91 (826.48–933.34)	0.96
CD8 + T (/ul)	825.85 (616.79–1034.91)	651.44 (598.89–703.98)	**0.07**
SP-A (ng/ml)	67.71 (60.64–74.78)	61.44 (60.03–62.85)	**0.06**
SP-D (ng/ml)	67.71 (60.64–74.78)	257.8 (253.45–262.14)	**0.02**
KL-6 (U/ml)	460.3 (408.44–512.16)	398.16 (381.49–414.83)	** < 0.01**
FVC (%)	67.52 (64.23–70.82)	68.56 (67.64–69.48)	0.52
Total	20	225	

Number in bold represents *P* < 0.05

ICI-P: immune checkpoint inhibitor related pneumonia; BMI: body mass index; PD-(L)1: programmed death (ligand) 1; COPD: chronic obstructive pulmoriary disease; IL-6: interleukin- 6; CRP: C-reaction protein; SP-A: sufactant protein A; KL-6: Krebs Von den Lungen-6; FVC: forced vital capacity

**Fig. 2:**
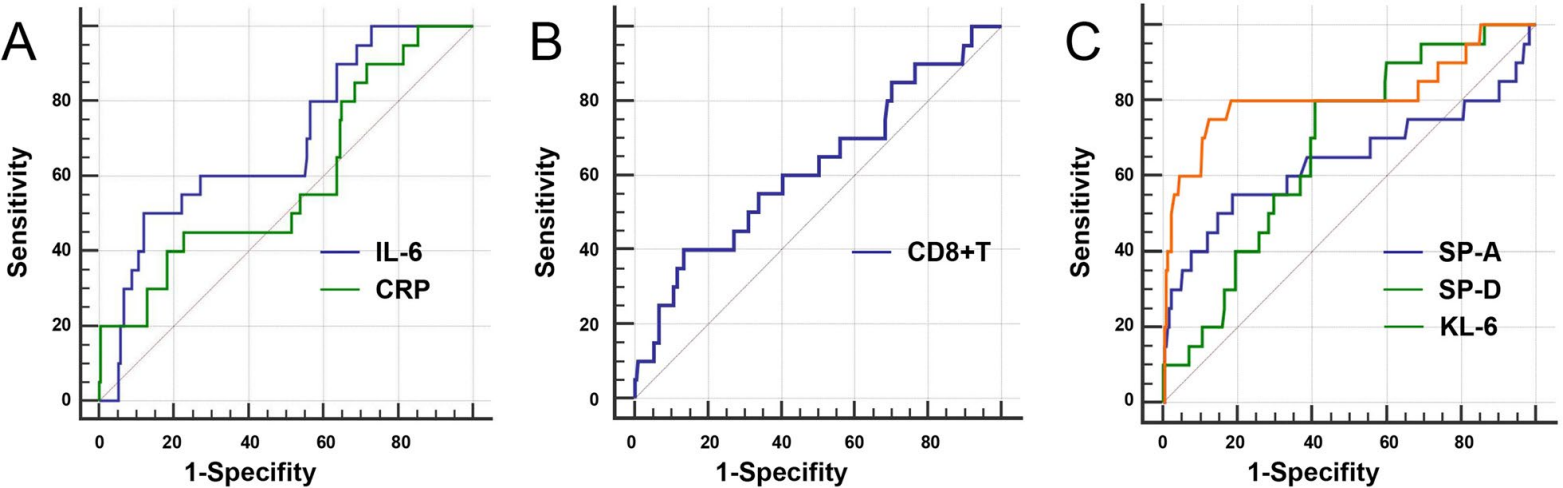
Diagnostic value of variables in immunotherapy-administrated patients. ROC of inflammatory markers (**A**, including IL-6 and CRP), CD8 + T Lymphocyte count (**B**) and serum alveolar protein (**C**, including SP-A, SP-D and KL-6)

**Fig. 3:**
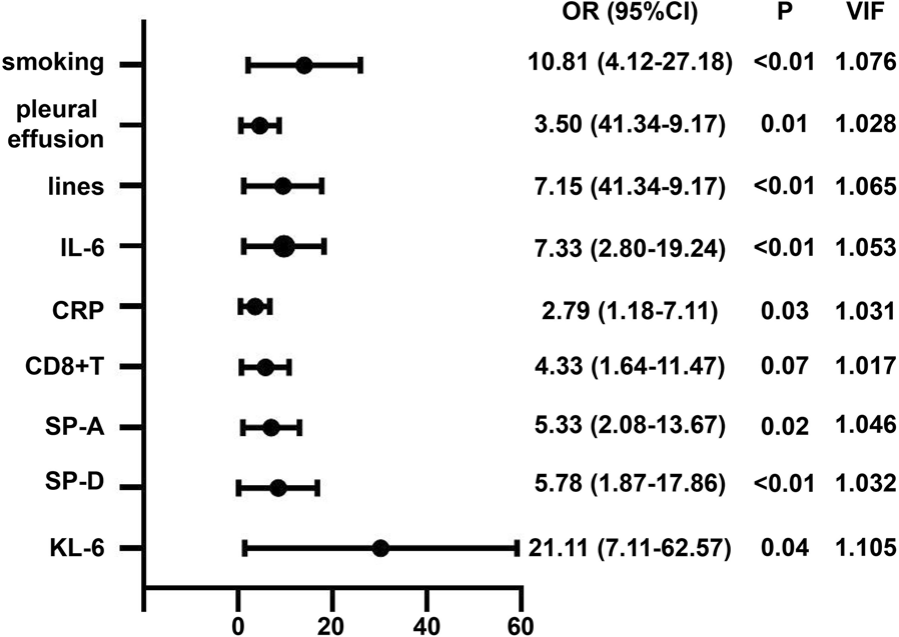
Forest plot of multivariate regression analysis for ICI-P. Horizontal axis: Odds ratio on a log scale with the reference line, odds ratio (circle) and 95% CI (whiskers)

All VIF values of the variables were close to 1, indicating no collinearity among the independent variables, and the normality test indicated that the variables were normally distributed, but Box–Tidwell analysis indicated that the relationships between the variables and CIP were nonlinear; therefore, we classified these continuous variables for logistic analysis. A ROC curve and the Youden index (sensitivity + specificity-1) were used to determine the optimal cut-off values of the variables due to the lack of a reference value for cancer patients in the recent literature. When CIP was defined as an endpoint, the optional cut-off values were listed (Table 3), and significant differences in the CIP rate were observed between the patients in the two groups (Fig. 2). By applying these cut-off values, the binary values were divided into two groups, and forest plots revealing the ORs between the two groups are shown in Fig. 3.

**Table 3:** Optional cut-off values of the variables

	Cut-off value	Sensitivity (%)	Specificity (%)	Youden index	AUC (95%CI)	*P*
IL-6	> 64.85 (pg/ml)	50.00	88.00	0.38	0.69 (0.63–0.75)	< 0.01
CRP	> 107.24 (mg/L)	45.00	77.33	0.22	0.59 (0.53–0.65)	0.14
CD8 + T	> 1106 (/ul)	40.00	86.67	0.27	0.62 (0.56–0.68)	0.09
SP-A	> 72.24 (ng/ml)	55.00	81.33	0.36	0.64 (0.58–0.70)	0.09
SP-D	> 267.79 (ng/ml)	80.00	59.11	0.39	0.68 (0.61–0.71)	< 0.01
KL-6	> 508 (U/ml)	75.00	87.56	0.63	0.81 (0.76–0.86)	< 0.01

*IL-6* interleukin-6, *CRP* C-reaction protein, *SP-A* sufactant protein A, *KL-6* Krebs Von den Lungen-6, *AUC* area under curve

### Binary logistic regression

Binary logistic regression analysis was performed for CIP risk based on clinicopathological features. The results showed that smoking history (OR = 168.56 95%CI 5.03–5643.91, *P* < 0.01), IL-6 (OR = 25.28 95%CI 1.9–335.89, *P* = 0.01), CRP (OR = 12.85 95%CI 1.21–137.02, *P* = 0.03), CD8 + T lymphocyte count (OR = 74.68 95%CI 4.09–1364.72, *P* < 0.01), SP-A (OR = 46.92 95%CI 2.54–867.77, *P* = 0.01) and KL-6 (OR = 123.44 95%CI 6.3–2420.57, *P* < 0.01) were independent influence factors of CIP.

### Nomogram construction

**Fig. 4:**
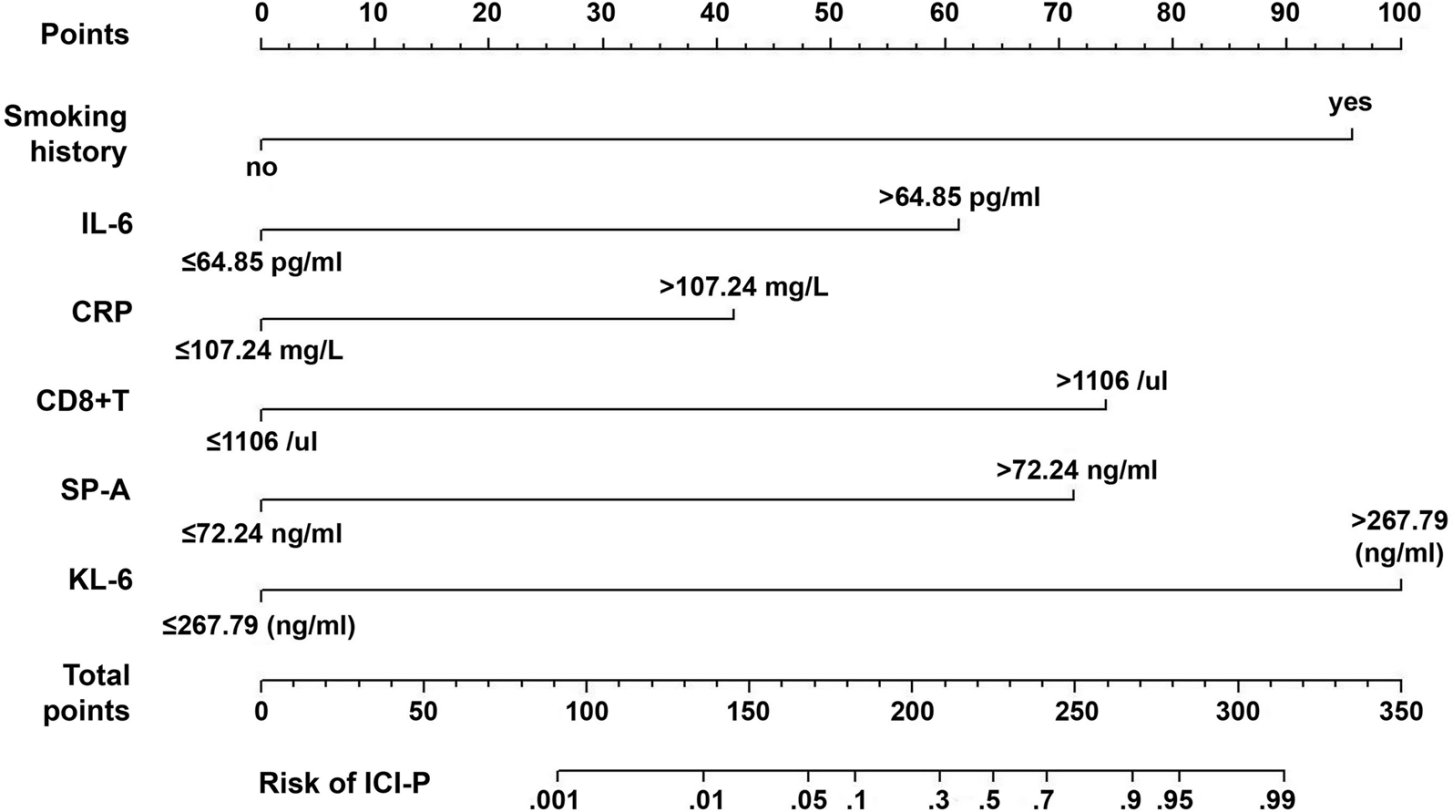
The establishment of clinical prognostic nomogram models to predict ICI-P risk

These independently associated risk factors were used to form CIP risk estimation nomogram (Fig. 4). The sum of points in the the bottom scales of the nomogram demonstrated the probability of CIP. The nomogram revealed that smoking history was the most influential prognostic factor, closely followed by KL-6 values. In addition, IL-6 values, CD8 + T lymphocyte count and SP-A values also made a moderate contribution to the survival outcome, while CRP value played minor roles.

### Nomogram evaluation

**Fig. 5:**
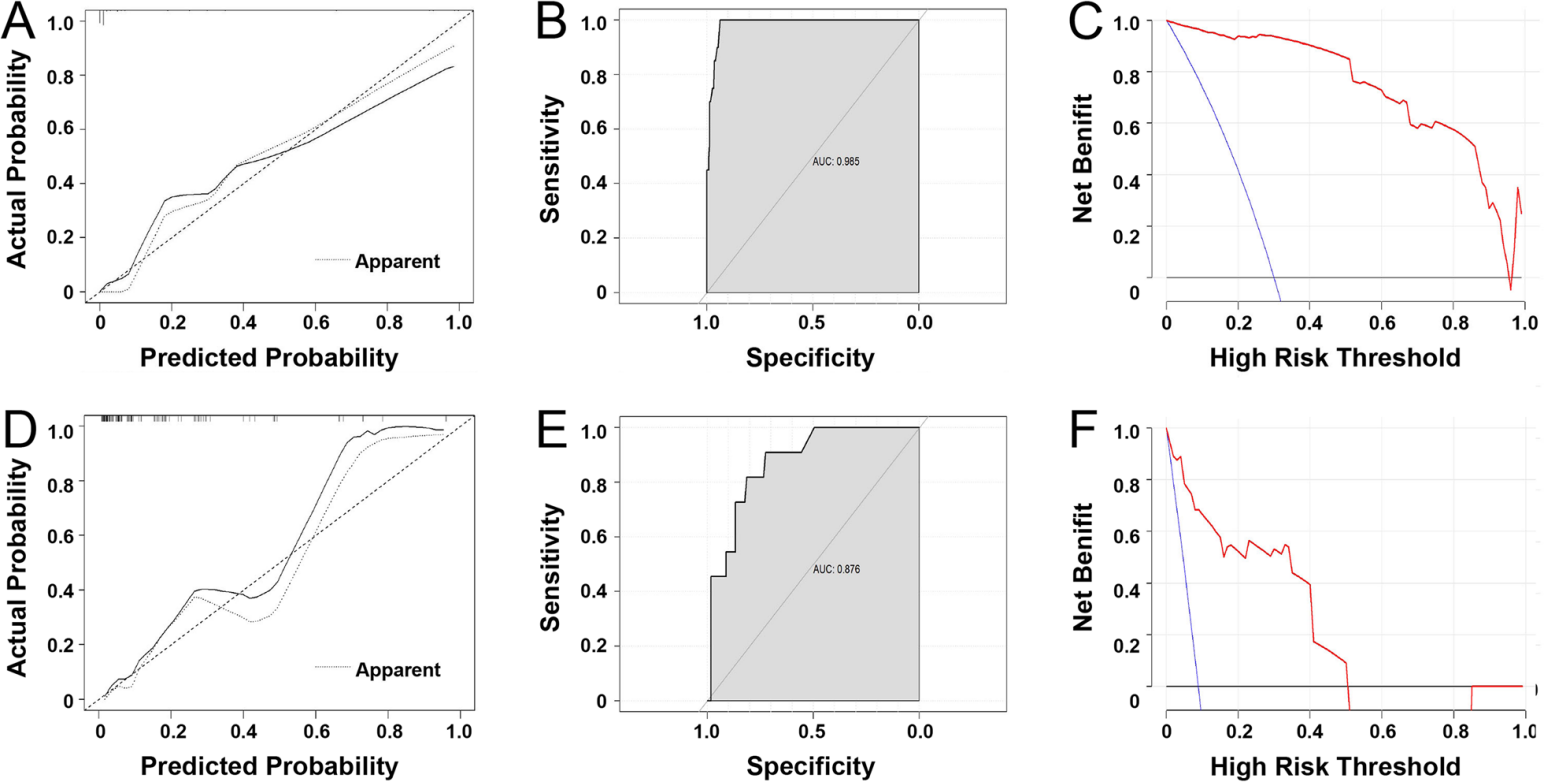
The evaluation of nomogram. **A**, **D** The calibration curves for predictions of ICI-P of training cohort (**A**) and validation cohort (**D**). The dashed line indicated ideal predictions, the solid line represents actual predictions of nomogram. The closer the distance of two lines, the better the performance of the predictive model. **B**, **E** ROC curves for the nomogram of training cohort (**B**) and validation cohort (**E**). The AUCs exceed 0.8, which demonstrated that the nomogram could predict the risk of ICI-P. **C**, **F** Decision curve analysis for the nomogram of training cohort (**C**) and validation cohort (**F**). The black line represents the net benefit at the time when no patients have ICI-P, while the blue line represents the net benefit at the time when all patients have ICI-P; the red line represents a model curve. The area under the three lines demonstrates the clinical usefulness of the nomogram

The excellent accuracy of the prediction value of the nomogram was also assessed by the calibration curves, and a preferable consistency between the nomogram-predicted and actual observed values was observed (Fig. 5A). As shown in Fig. 5B, the AUC value of the nomogram for CIP in the training cohort was 0.98 (0.994–1.00), which was higher than the AUC values of any variables alone. Finally, DCA was conducted to assess the clinical utility of the nomogram, and the nomogram showed good consistency in forecasting the CIP risk at all thresholds in the training cohort, implying the good capability of our model (Fig. 5C).

To further evaluate the nomogram, we assessed its predictive performance in the validation cohort (Fig. 5D–F). The calibration curve in the validation cohort indicated that the model had good discrimination, and the AUC of the nomogram was 0.88 (0.73–0.91). In addition, the DCA illustrated that the nomogram had favourable potential clinical applicability in predicting the CIP risk in the validation cohort, with a 0–0.5 threshold probability. In conclusion, validation of the nomogram showed a good level of agreement with the predictive value.

## Discussion

Biomarkers are usually defined as changed indicators of objectively measured physiological/pathological processes or pharmacological responses to therapeutic interventions [26], which play an important role as a tool for differential diagnosis, prognosis and monitoring disease progression. A nomogram is an intuitive tool for assessing the individual probability of a clinical event based on a statistical predictive model [27], which is also used to predict the risk of adverse events related to tumour treatment [28, 29]. However, few studies have reported irAE-related nomograms, especially for cancers other than lung cancer [30]. In this study, we established a nomogram to predict the risk of CIP according to the baseline clinical characteristics and laboratory values.

The definition of CIP is the occurrence of respiratory symptoms/signs associated with a new emerging infiltration on chest imaging [31]. The causes of CIP include damage to AECs by inflammatory cytokines and activated CD8 + T cells and the recognition of some preexisting antigens (such as ANCA) on AECs by the PD-(L)1 monoclonal antibody. In all kinds of cancers, the overall incidence of CIP varies from 3 to 5% for all grades [32], which is slightly lower than that in our study. The main reasons for include the following: in our study, routine HRCT was used, and more patients were treated with posterior-line therapy.

IL-6 was found in 1973 as a soluble factor that is secreted by T cells and it has been shown to be involved in T-cell activation and the induction of cytokines. CRP was initially found to be a serum protein and it is synthesized in the liver, and its production could be stimulated by IL-6 [33]. IL-6 and CRP are proinflammatory cytokines involved in immune cell recruitment, proliferation and effector functions. IL-6 plays a key role in the systemic inflammatory response, and the IL-6 receptor antagonist tocilizumab showed clinical improvement of a wide variety of irAEs [34]. Previous studies reported that IL-6 and CRP elevation could predict irAEs, especially CIP [35, 36]. We also found that elevated IL-6 and CRP were independent risk factors for CIP, and IL-6 accounted for a larger proportion.

The significant upregulation of activated CD8 + T lymphocytes by ICIs might be the first trigger and it plays a vital role in the occurrence and development of irAEs. The local infiltration of CD8 + T lymphocytes is an important cause of irAEs. Zhou et al. found that the higher the proportion of CD8 + T cells in lung cancer tissue was, the higher the probability of CIP [37]. In addition, the local infiltration of CD8 + T lymphocytes in the skin and hair follicles was also related to immunotherapy-related psoriasis, such as dermatitis [38] and alopecia [39]. Wang et al. also found that pretreatment absolute lymphocyte count was related to an increased risk of irAEs [40]. However, there is still no report on the relationship between lymphocyte taxonomic count and irAEs. In this study, we found elevated CD8 + T lymphocytes in the peripheral blood in CIP patients, suggesting that lymphocyte infiltration may play an important role in CIP.

Radiographic features are currently important determinants for the diagnosis, severity and prognostic assessment of CIP; however, certain serum protein expression levels are also correlated with the severity of CIP and may serve as biomarkers for determining the clinical aspects of CIP. These serum proteins include alveolar epithelial proteins (including surfactant protein and KL-6), chemokines and cytokines (including CCL18, CCL2, CXCL10 and YKL-40) and MMPs and tissue inhibitors of MMPs. In our study, we found that serum alveolar epithelial proteins before treatment could predict CIP risk, but unfortunately, we were not able to study other serum proteins in predicting CIP. A previous study showed that changes in KL-6 levels are correlated with interstitial pneumonia caused by CTD [41]. Therefore, the relationship between serum alveolar epithelial proteins and the efficacy of glucocorticoids in the treatment of CIP needs to be further studied.

Many factors can damage AECs, such as preexisting pulmonary diseases, including chronic obstructive pulmonary disease (COPD), asthma and pleural effusion [32], and some anticancer treatments, such as chemotherapy [42], targeted therapy [43] and radiotherapy [44]. In this study, we found that smoking history and pleural effusion were associated with CIP, while smoking history was an independent risk factor for CIP. Smoking history, which is easily available information, could be used as a guide in clinical practice for irAE prediction.

There are also many shortcomings of this study. First, this study is a dual-centre study from northeastern China, and there may be inclusion bias in the process of recruiting patients. Second, patients with CIP failed to undergo bronchoalveolar lavage or lung biopsy, which helps to clarify the specific lesions of CIP, especially the infiltration of immune cells. Finally, the sample size of this study was small, and no CTLA-4 inhibitors were used by the included patients. We look forward to a large-scale, multicentre prospective study to explore additional irAEs, especially CIP-related markers.

## Conclusion

Smoking history, acute phase proteins (IL-6 and CRP), CD8 + T lymphocyte count and serum alveolar epithelial proteins (SP-A and KL-6) are the risk factors of CIP. By combining risk factors, nomograms were constructed for CIP. The models provide an early prediction method for CIP before ICI administration, which facilitates early diagnosis and rational treatment.

## Data Availability

All data generated or analyzed in this study (with patient information hidden) can be obtained from the corresponding authors upon reasonable request.

## Abbreviations

AECs: Alveolar epithelial cells
AUC: Area under the curve
BMI: Body Mass Index
BSA: Bovine serum albumin
CIP: Immune checkpoint inhibitors pneumonia
COPD: Chronic obstructive pulmonary disease
CTD: Connective tissue disease
DCA: Decision curve analysis
EGFR-TKIs: Epidermal growth factor receptor-tyrosine kinase inhibitor
ELISA: Enzyme linked immunosorbent assay
GGA: Ground-glass attenuation
HRCT: High resolution computed tomography
ICIs: Immune checkpoint inhibitors
irAEs: Immunotherapy related adverse events
KL-6: Krebs von den Lungen-6
OD: Optical density
PD-(L)1: Programmed Death-(Ligand) 1
ROC: Receiver operating characteristic
SP-A: Surfactant protein A
VIF: Variance inflation factor
